# Conversion of a rice CMS maintainer into a photo- or thermo-sensitive genetic male sterile line

**DOI:** 10.1007/s11032-018-0805-2

**Published:** 2018-04-18

**Authors:** Yanning Tan, Xuewu Sun, Baohua Fang, Dong Yu, Zhizhong Sun, Weiping Wang, Xiabing Sheng, Xiaolin Yin, Ling Liu, Yongfei Zhang, Meijuan Duan, Dingyang Yuan

**Affiliations:** 1 State key Laboratory of Hybrid rice, Hunan Hybrid Rice Research Center, Changsha, 410125 China; 2 Hunan Academy of Agricultural sciences, Changsha, 410125 China; 3 Hunan Rice Research Institute, Changsha, 410125 China; 4 College of Bioscience and Biotechnology, Hunan Agricultural University, Changsha, 410128 China; 5 Longping Branch of Graduate School, Hunan University, Changsha, 410125 China; 6 Hunan agricultural Biotechnology Research Center, Changsha, 410125 China

**Keywords:** Hybrid rice, Heterosis utilization, Maintainer line, Photoperiod/temperature-sensitive male sterile line, ^60^C_O_-γ irradiation

## Abstract

A maintainer line of 3-line hybrid rice commonly presents a certain genetic distance to a 2-line restorer line, but in many cases, 2-line restorer lines present defects upon recovery of the object cytoplasmic male sterile (CMS) line of the maintainer line, which impedes the utilization of their heterosis. Here, we report a strategy and an example of converting a maintainer into a photoperiod/temperature-sensitive genic male sterile (P/TGMS) line with an almost identical genetic background, thus maximizing the heterosis. Firstly, through treatment of maintainer line T98B with ^60^C_O_-γ irradiation, we identified the TGMS line T98S, which is sterile at higher temperatures and fertile at lower temperatures. Secondly, the T98S line was proven to be identical to T98B with regard to genetic background via an examination of 48 parental polymorphous SSR markers and exhibited excellent blossom traits similar to those of T98B, with an extensive forenoon flowering rate of 75.92% and a high exertion rate of 64.59%. Thirdly, in a combination test, three out of six hybrids from T98S crossed with 2-line restorer lines showed a yield increase of 6.70–15.69% for 2 consecutive years. These results demonstrated that the strategy can generate a new P/TGMS line with strong general combining ability (converted from a maintainer line), thus helping to increase the genetic diversity of male sterile heterotic groups.

**Electronic supplementary material:**

The online version of this article (10.1007/s11032-018-0805-2) contains supplementary material, which is available to authorized users.

## Introduction

It is of great importance to breed high-yield hybrid rice to feed the growing worldwide population. The 3-line and 2-line systems are the two basic methods employed to generate hybrid rice seeds. These two methods differ in breeding efficiency due to their use of two different types of male sterile lines. The 3-line system utilizes a cytoplasmic male sterile (CMS) line, whose multiplication is managed by using a maintainer line. Usually, the CMS line and its corresponding maintainer are genetically very close siblings, differing only in the loci responsible for controlling flower and pollen behavior. In the production of F_1_ hybrids, the male sterility conferred by the CMS line is restored by a so-called restorer line (R line), as male sterility is controlled by cytoplasm and nucleus interaction, with the nuclear R gene being dominant over the cytoplasmic CMS gene (Yuan et al. 2003). Less than 5% of current germplasm resources can be used as restorer lines for 3-line system hybrid production, which makes it difficult to obtain elite combinations (Yuan 1997; Huang et al. 2014).

In contrast, the 2-line system is based on a photoperiod/temperature-sensitive genic male sterile (P/TGMS) line. Because the P/TGMS line is controlled by a recessive nuclear gene(s), almost any normal cultivar can be used as its restorer line, thus greatly improving the probability of selecting favorable combinations (Yuan 1997). In addition, the 3-line and 2-line systems vary in terms of the propagation of their sterile lines. The P/TGMS line can self-reproduce if the environmental photoperiods and/or temperature conditions are favorable (Yuan 1997). Such advantages as a broad selection of restorer lines, relative freedom to combine with male lines and a simplified procedure for propagating the sterile line make the 2-line system superior in hybrid rice breeding, enabling breeders to realize higher heterosis and explore commercial breeding of heterotic groups more efficiently.

However, developing high-yield hybrid rice is still very challenging because heterosis in rice is not yet fully understood. Heterosis in hybrid rice relies on parental distance and generally, the greater the distance, the stronger the heterosis (Ding et al. 2007). In particular, parental lines of different geographic origins, of different ecotypes or belonging to separate subspecies, are likely to generate combinations with strong heterosis. For instance, the 3-line hybrid rice Shan-You 63 was derived from two cultivars with a large geographical distance between their areas of origin—Zhen-Shan 97A/B—which is an early-season rice planted along the Chinese Yangtze River, and Ming-Hui 63, an inbred line created from the Philippine rice IR630 and the Guyana rice Gui-630 (Xie et al. 1987). Another example is the 2-line hybrid rice Liang-You-Pei 9, which is the offspring of the Indica-Japonica male sterile line Pei-ai 64S and the Indica restorer line 93-11 (Lu and Zou 2000). Genetic diversity among cultivars is declining because a limited number of key lines have been used widely and repeatedly. Therefore, developing a reasonable solution to extend the parental genetic distance is critical in pushing genetic gains in hybrid rice breeding.

The 3-line maintainer line commonly presents a certain genetic distance to a 2-line restorer line, which provides a considerable opportunity to generate P/TGMS lines by converting the maintainer line. Maintainers with a strong general combining ability and desirable blossom habits are ideal donors for P/TGMS lines. At present, the most successful P/TGMS line Y58S originated from the maintainer line V20B and Fei-Gai B (Deng 2005). To date, more than 90 hybrids with Y58S as a parental line have been released (http://www.ricedata.cn/variety/). A recent study identified a total of 20,562 non-synonymous SNPs spanning 8854 genes between maintainer line V20B and 2-line restorer line 93-11 through deep re-sequencing (Hu et al. 2014), revealing that a considerable genetic distance existed between the maintainer line and the 2-line restorer line.

The P/TGMS line in release is primarily composed of two types: the PTGMS line and TGMS line (Chen et al. 2010). The fertility of the PTGMS line is co-regulated by photoperiod and temperature, while that of the TGMS line is controlled only by temperature (Cheng et al. 1996). The PTGMS trait and TGMS trait are independently controlled by the *p/tms12-1* (or *pms3*) and *tms5* genes (Zhou et al. 2012; Ding et al. 2012; Zhou et al. 2014). Hence, breeders have transferred these genes into other genetic backgrounds to generate new P/TGMS lines, but this work is labor-intensive and requires more than 5–6 years.

Here, we proposed a strategy for converting the maintainer line into a practical P/TGMS line with an identical genetic background with the exception of the mutated locus, thus ensuring that 2-line restorer lines can be utilized with P/TGMS lines for developing high-yield combinations through mutagenesis. In this study, we report the successful conversion of the maintainer line T98B into a P/TGMS line (T98S) via gamma irradiation treatment. The logic is depicted in Fig. S1. The agronomic traits of T98S and its 2-line-based hybrid rice were also studied.

## Materials and methods

### Materials and experiment site

The rice variety T98B is an Indica maintainer line developed from wild-abortive CMS line T98A. T98S is a P/TGMS line acquired from T98B via ^60^C_O_-γ irradiation. The six early-season Indica cultivars used, including Xiang-Zao 45 (XZ45), Zhong-Jia-Zao17 (ZJZ17), Zao-116 (Z116), Zao-102 (Z102), Zao-143 (Z143), and Zao-996 (Z996), are conventional varieties or 2-line restorer lines. All of the above materials were provided by the State Key Laboratory of Hybrid Rice, Changsha, Hunan Province, China.

The radiation treatment of T98B was contracted to Hunan Provincial Radiation Center, Changsha, China, in 2010. Approximately 9000 mature seeds from T98B were treated with ^60^Co-γ at 300 Gy (5.5 Gy/min for 55.5 min). Then, the M_0_ seeds were germinated and transplanted singly in a field in Changsha, China. The screening of P/TGMS plants in the field was conducted in Sanya, Hainan Province, and Changsha, Hunan Province, China, from 2011 to 2012. The verification of the TGMS trait in T98S in-chamber was performed in Changsha in 2013. The field investigation of the basic agronomic traits of T98S was carried out in Changsha in 2013 and 2014, blossom traits were examined in Changsha in 2014, and the yield components were examined in Changsha in 2015 and 2016. Differences among the lines were analyzed using a *t* test at 0.05 and 0.01 with one-way ANOVA.

### Identifying P/TGMS lines

After transplanted M_0_ plants reached maturity, mixture harvesting was conducted to obtain 10–15 seeds per plant. Then, an M_1_ population was planted with nearly 25,000 single plants in the Sanya winter nursery, and screening of sterile plants was conducted in April with 3% I_2_-KI using an optical microscope. These sterile plants were then regenerated in Changsha to identify P/TGMS mutants between May and October. Seeds were gathered from the candidate P/TGMS plants following fertility observation in the growth chamber.

At phase IV of young panicle differentiation, the candidate P/TGMS plants were treated in the growth chamber under four fixed conditions, which were adjusted to low temperature at 22.0 °C (20/24 °C) and high temperature at 28.0 °C (26.0/30.0 °C) combined with a short photoperiod of 12.0 h and a long day length of 13.5 h. Ten plants were subjected to each treatment, and each plant was investigated for fertility to identify the factor by which it was influenced.

### Genetic authenticity test and genetic characterization analysis

Evaluation of the genetic difference between T98S and T98B was performed by examining 48 parental polymorphous SSR markers following the standards of NY/T 1433-2014 formulated by the Ministry of Agriculture of P. R. China. DNA was extracted from the leaves of T98S and T98B using the CTAB method (McCouch et al. 1988); after which, the DNA samples were amplified. The PCR reaction system was 12.0 μL in volume, including 5.0 μL of H_2_0, 5.0 μL of 2 × Easy Taq PCR Super Mix (TransGen, Beijing, China), 1.0 μL of 10 μM primers and 1.0 μL template DNA. The PCR program was run at an annealing temperature of 55–63 °C for 32 cycles. Its products were visualized with PAGE gel electrophoresis.

To study the genetic behavior of T98S, two crosses of T98S with normally fertilized cultivars T98B and XZ45 were generated. Later, the fertility of the F_1_ and F_2_ groups was investigated under high-temperature conditions from July to August (with a mean daily temperature above 32.0 °C) in Changsha, China. Summation of the number of TGMS plants and wild-type plants for each F_2_ group was conducted to deduce the genetic pattern of T98S with χ^2^_0.05_ = 3.84.

### Agronomic characteristic analysis

T98S seeds were sown in two batches (one in late March and one in late May) and transplanted in three replicates (each replicate contained 50 plants), then compared with T98B and T98A. Five successive plants were selected from every replicate to measure and analyze the basic agronomic characteristics, including the days from sowing to heading, plant height (PH), panicle length, effective panicles per plant (EPP), grain number per panicle (GNP), and seed-setting rate (SSR).

### Blossom habit analysis

At flowering, five panicles from T98S, T98B, and T98A were selected for marking blossomed flowers every 30 min from 6:00 a. m. to 18:00 p. m. in order to calculate the flowering rate intervals of the three replications. The mean flowering rate was calculated for each line. We also separately collected blossomed flowers from five panicles of T98S, T98B, and T98A over the three replicates to record and compare the percentage of flowers with exerted stigma.

### Combinations test

T98S and T98A were crossed with six cultivars as restorer lines, including XZ45, ZJZ17, Z116, Z102, Z143, and Z996. Hybrid seeds from all combinations, with Zhu-Liang-You 819 (ZLY819) as a control, were randomly arranged in the same field over three plots, and each plot contained 100 plants. From every plot, five successive plants were selected to investigate the basic agronomic characteristics, including whole growth period (WGP), PH, EPP, GNP, SSR, and thousand-grain weight (TGW), and every plot was harvested to collect data on the yield per plant (AYP).

## Results

### T98S obtained from maintainer line T98B through irradiation and confirmed as a TGMS line

**Fig. 1:**
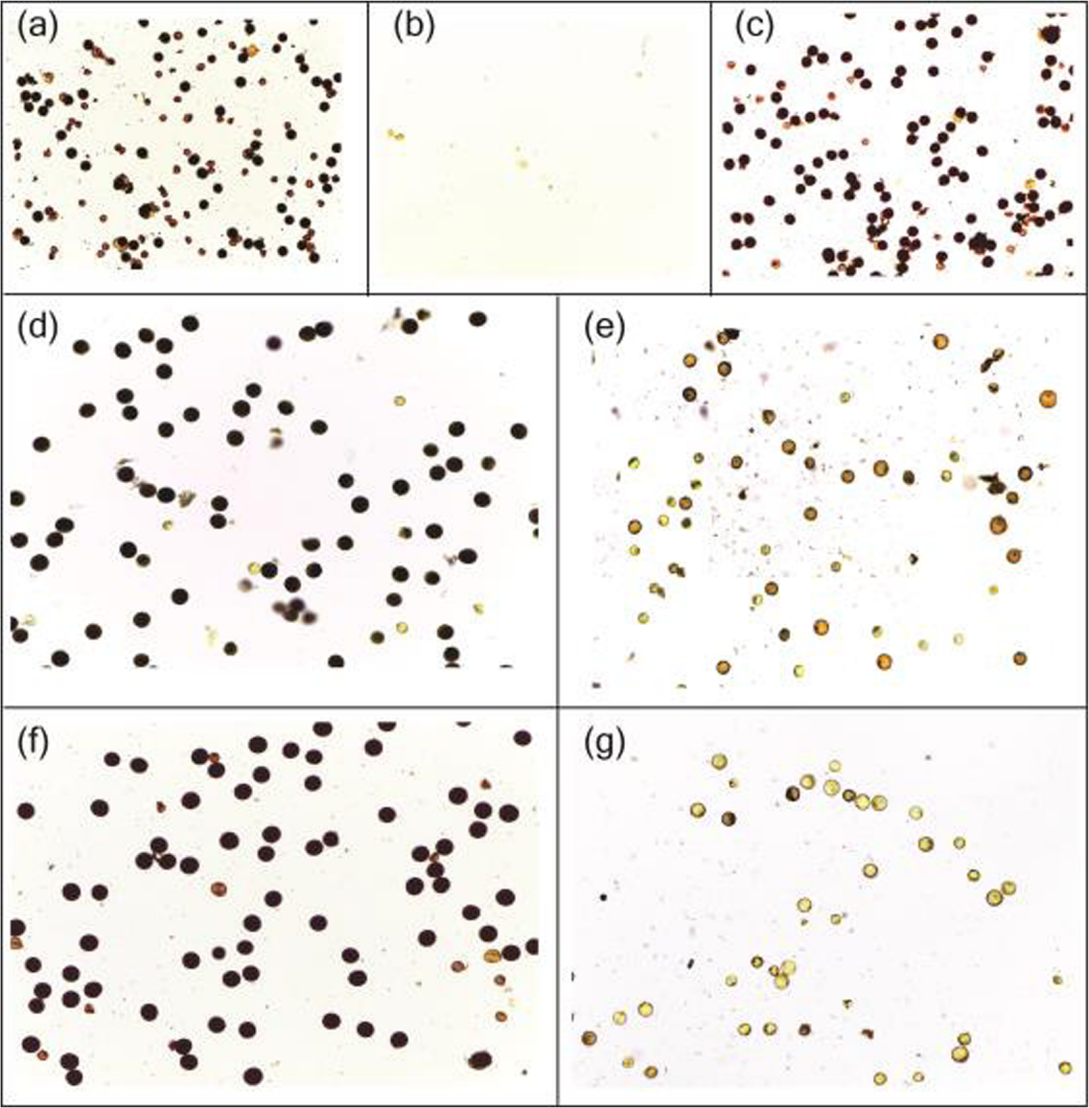
T98S showed a P/TGMS trait in-field and in-chamber. Fertility observations on May 6th (**a**), July 12th (**b**), and September 25th (**c**) in the field, and fertility performance under 22.0 °C/12.0 h (**d**), 28.0 °C/12.0 h (**e**), 22.0 °C/13.5 h (**f**), and 28.0 °C/13.5 h (**g**) in-chamber

Among 9000 seeds treated by gamma irradiation, a total of 5200 M_0_ seeds germinated normally, and 76 plants were found to be sterile in the M_1_ population when headed in April in a winter nursery in Sanya, China (with a monthly mean temperature of approximately 28.5 ± 3.4 °C and a photoperiod of 12.5 ± 0.2 h). Later, two regenerated plants labeled BM2-10 and BM2-23 were observed to change in fertility from May to October when planted in Changsha, China. If headed before May 10th, in accordance with a daily mean temperature below 25 °C and a photoperiod of approximately 13.10–13.20 h during the young panicle differential phase, the two plants presented light yellowish anthers and accumulated a few round-and-stained normal pollen grains (Fig. 1a). If headed from early June to early September, when the daily mean temperature ranged from 28.0 to 34.0 °C and the photoperiod ranged from 12.0 to 13.7 h, they exhibited white shrunken anthers and maintained a sterile state. In this phase, the plants presented three types of abortive pollen grains, namely, round-but-empty pollen, irregular pollen, and null pollen, and the null pollen grains occurred at the highest temperature from June 20th to August 15th (Fig. 1b). Thereafter, with the temperature and photoperiod declining from September 25th to October 20th, a conversion from sterility to fertility was observed (Fig. 1c). Based on the definition of P/TGMS, we supposed that BM2-10 and BM2-23 would be P/TGMS plants. Since the two plants were later identified as allelic (data not shown), we refer to BM2-10 as T98S for convenience in this study.

To clearly determine the factors to which T98S would be sensitive, a growth chamber test under fixed conditions was employed. T98S was fertile at 22.0 °C and below, but the line was sterile at 28.0 °C and above, no matter the photoperiod, which was adjusted to 12.0 or 13.5 h (Fig. 1d–g). In particular, there were approximately 70.0–80.0% round-and-stained normal pollen grains and less than 30.0% abortive pollen grains at 22.0 °C, but there were nearly 100.0% abortive pollen grains at 28.0 °C under the same photoperiod (Fig. 1d–g), indicating that temperature affected the degree of pollen sterility. These data from field observation and growth chamber observation demonstrated that T98S is a TGMS line.

### T98S was shown to be identical to T98B in genetic background and inheritance via a recessive nuclear gene

**Fig. 2:**
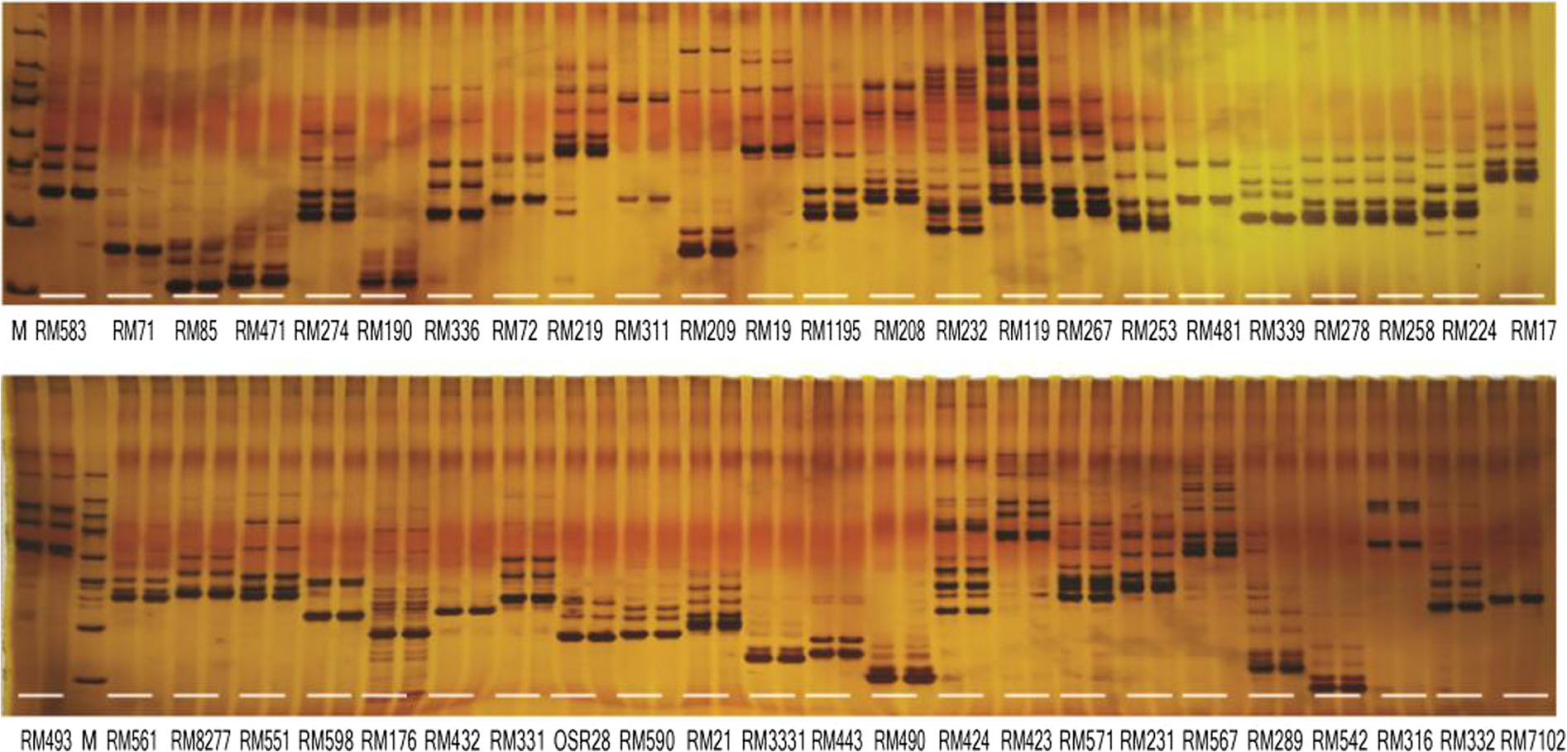
Detection of the genetic backgrounds of T98S and T98B using 48 parental polymorphous SSR markers. T98S and T98S presented indistinguishable genotypes for each polymorphous SSR marker. Each marker was used to test two samples, with the first being T98B and the second being T98S. M, 50 bp DNA ladder

In a genetic authenticity test and genetic characterization analysis using SSR markers (Lin et al. 2016), a total of 48 parental polymorphous SSR markers were used to primarily evaluate the genetic difference between T98S and T98B. The two lines were found to yield indistinguishable fingerprinting patterns, indicating that T98S is essentially derived from T98B, and the present authenticity test recommended by NY/T 1433-2014 formulated by the Ministry of Agriculture of China is unable to distinguish them from each other (Fig. 2). Furthermore, the two F_1_ populations based on T98S hybridized with wild-type cultivars T98B and XZ45 were both fertile under high-temperature conditions. However, in their F_2_ population, the lines displayed two phenotypes in fertility, including TGMS and the wild type with a theoretical separation ratio of 1:3, indicating that the T98S phenotype is conferred by a recessive nuclear gene mutation alone (Table S1).

### T98S presented a similar shape to T98B and showed the same agronomic traits as T98A

At M_2_, T98S showed a similar shape to T98B with tight branches, long flag leaves, slender caryopsis, colorless apiculus, and colorless stigma (Fig. S2). Agronomic trait observations suggested that T98S was an early-season TGMS line whose flowering is sensitive to temperature, as has been observed with T98B. If sown on March 28th, T98S would undergo heading after 74 days, but when sown on May 20th, it would advance to head in 12 days. Naturally, the sterility of T98S caused a heading delay of 4–6 days and a decrease of 13.6–15.5 cm in plant height, but it exhibited an increase of 21.25–25.92% in the tiller number compared with that of T98B. However, T98S and T98B presented the same panicle lengths and grain number per panicle. Interestingly, if T98A (a CMS line of T98B) was regarded as a control, there was nearly no difference in plant shape, growth period, and yield factors (Table 1).

**Table 1:** Comparison of basic agronomic characteristics among T98S, T98B, and T98A

Year and location	Lines	Sowing date (month/day)	Days from sowing to heading	Plant height (cm)	Panicle length (cm)	Effective panicle per plant	Grain number per panicle	Seed-setting rate (%)
2013 Changsha, China	T98S	3/28	74.6 ± 1.1a	76.4 ± 2.1ab	23.6 ± 0.6a	10.2 ± 0.9a	136.7 ± 3.9a	1.3 ± 0.9ab
	T98B	3/28	69.3 ± 0.6ab	91.5 ± 1.7a	24.3 ± 0.8a	8.1 ± 0.6ab	141.5 ± 2.9a	84.6 ± 1.6a
	T98A	3/28	75.3 ± 0.5a	77.4 ± 1.9ab	23.8 ± 0.7a	10.4 ± 0.7a	132.5 ± 3.4ab	0.0 ± 0.0ab
	T98S	5/20	62.0 ± 1.0a	74.8 ± 2.0ab	21.7 ± 0.5a	9.2 ± 0.8a	123.9 ± 3.1ab	0.0 ± 0.0ab
	T98B	5/20	57.6 ± 0.6ab	88.4 ± 1.8a	22.4 ± 0.7a	7.6 ± 0.6ab	131.6 ± 3.3a	82.2 ± 2.7a
	T98A	5/20	61.3 ± 0.6a	76.3 ± 1.4ab	21.6 ± 0.5a	9.9 ± 0.9a	121.6 ± 2.6ab	0.0 ± 0.0ab
2014 Changsha, China	T98S	3/25	73.3 ± 0.6a	80.5 ± 2.6ab	24.3 ± 0.7a	10.7 ± 0.6a	121.7 ± 4.1a	2.2 ± 1.1ab
	T98B	3/25	68.6 ± 1.1ab	94.5 ± 2.1a	25.2 ± 0.9a	8.5 ± 0.7ab	125.5 ± 4.7a	81.3 ± 1.3a
	T98A	3/25	74.3 ± 0.5a	78.4 ± 1.7ab	24.7 ± 0.8a	10.4 ± 0.8a	127.5 ± 5.2a	0.0 ± 0.0ab
	T98S	5/24	58.3 ± 0.6ab	77.8 ± 1.9ab	23.1 ± 0.7a	9.9 ± 0.8a	134.7 ± 5.6a	0.0 ± 0.0ab
	T98B	5/24	52.6 ± 0.6ab	93.3 ± 2.6a	23.6 ± 0.8a	7.9 ± 0.6ab	136.2 ± 4.9a	78.5 ± 3.4a
	T98A	5/24	59.6 ± 0.6a	78.1 ± 2.1ab	22.9 ± 0.9a	10.2 ± 0.6a	131.2 ± 4.5a	0.0 ± 0.0ab

Values are mean ± SD (*n* = 5). The data followed by the same letters presented no significant difference at *p* < 0.05

### T98S inherited the excellent blossom habits of T98B

**Fig. 3:**
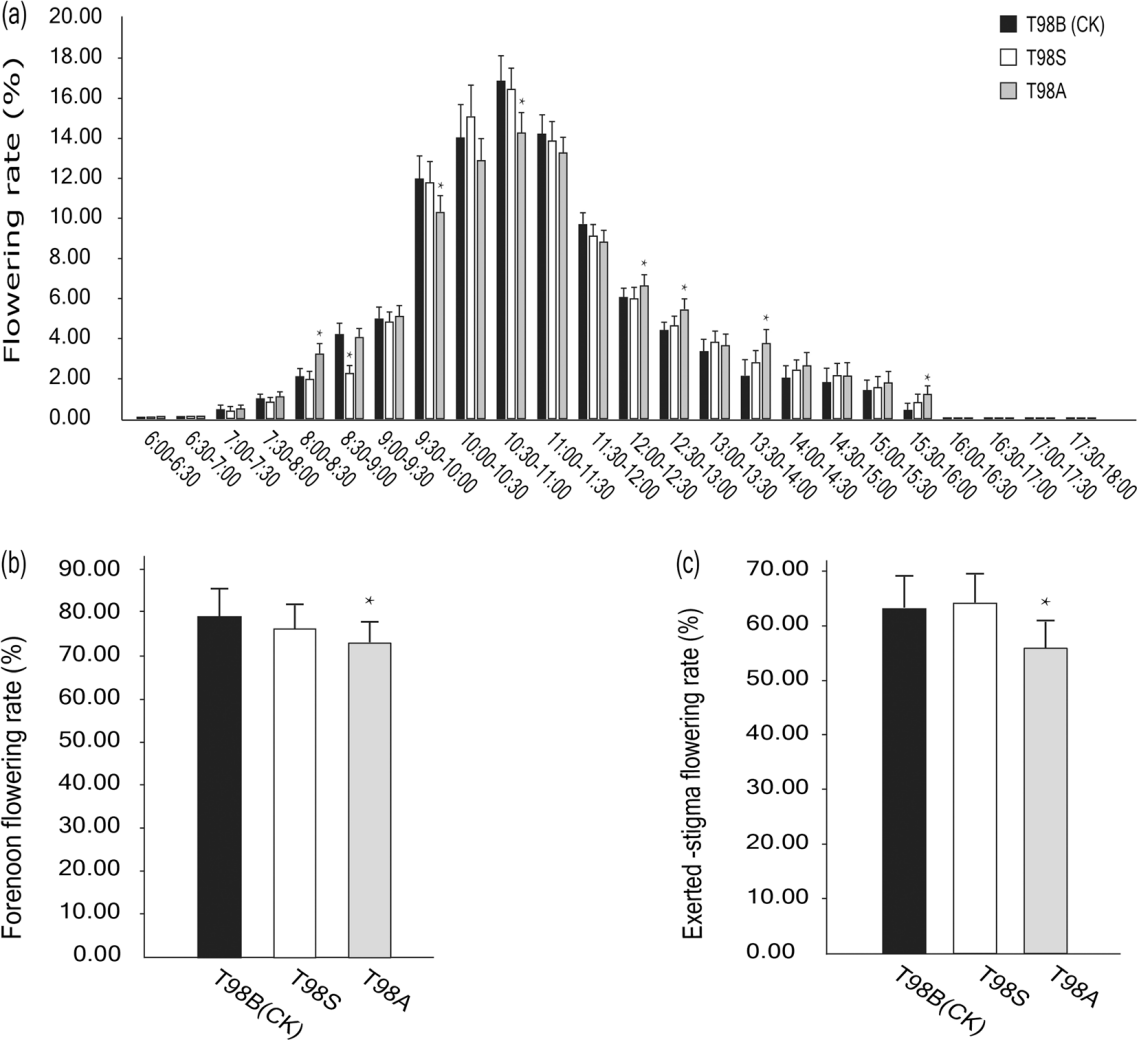
Comparison of flowering habit traits among T98S, T98A, and T98B. Asterisk (*) indicates a significant difference at *p* < 0.05

The chart of daily flowering dynamics showed that T98S, T98B, and T98A exhibited almost the same, if not identical, flowering behavior. T98S began to flower at 7:00 a.m., kept its climax from 9:30 to 12:00, and ended before 16:00 p.m. (Fig. 3a). In hybrid seed production, a forenoon flowering rate (before 12:00 a.m.) and an exerted-stigma flower rate are considered key indicators closely related to productivity. T98S performed well in these traits, reaching a forenoon flowering rate of 75.92% and a high stigma exertion rate of 64.59% (Fig. 3b, c). Interestingly, T98A showed a lower forenoon flowering rate and exerted-stigma flower rate (Fig. 3b, c). One possible reason for this difference was that the cytoplasmic sterility of the CMS line harmed its tolerance for high temperatures.

### Combinations based on T98S crossed with 2-line restorers showed yield potential

**Fig. 4:**
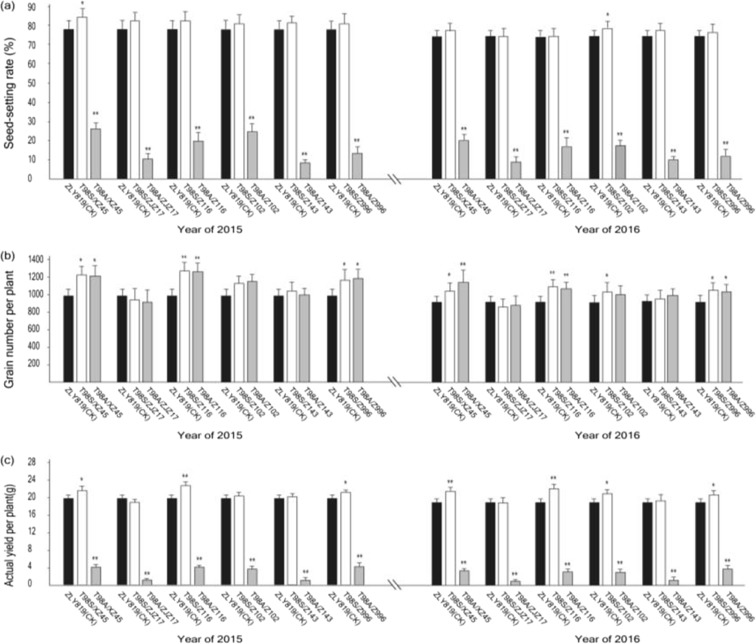
Evaluation of combinations via a main yield factor. The combination test of T98S and T98A crossed with the same group of restorer lines. (**a**) Seed-setting rate. (**b**) Grain numbers per plant. (**c**) Actual yield per plant. All combinations with T98S show a normal SSR and AYP, while the combinations with T98A were defective in these aspects, but grain number per plant is less likely affected when comparing T98S and T98A. Single and double asterisks indicate *p* < 0.05 and *p* < 0.01, respectively

In combination testing, TGMS line T98S and CMS line T98A were crossed with the same restorer line. The two hybrids showed similar agronomic yield factors, including PH, WGP, EPP, GNP, and TGW, through field testing for two consecutive years (Table S2), suggesting that the performance of T98S is identical to that of T98A. Six restorer lines (XZ45, ZJZ17, Z116, Z102, Z143, and Z996) were all proven unrecoverable by the wild-abortive CMS line T98A according to the seed-setting rate (SSR) of their combinations (8.20–26.32% in 2015 and 7.55–20.02% in 2016). Meanwhile, the corresponding combinations with T98S were all normally fertilized, harboring a seed-setting rate of 81.31–84.47% in 2015 and 73.53–77.74% in 2016, which was slightly more or less, respectively, than that of the control ZLY819 at 78.03% in 2015 and 73.50% in 2016 (Table S2; Fig. 4a).

Compared to ZLY819, the combinations exhibited superior GNP, which ensured that almost all combinations except for T98S/ZJZ17 exceeded the control in grain number per plant (GNP × EPP), despite weakness in EPP for T98S/Z102 and T98S/Z996. Particularly, three combinations (T98S/XZ45, T98S/Z116, and T98S/Z996) showed a significant change in GNP (Table S2; Fig. 4b). The TGW was decreased in T98S/Z102 and increased in T98S/ZJZ17, while it remained nearly the same in the other combinations (Table S2).

The AYP reflects the real performance of tested hybrids. Due to unfavorable high-temperature conditions, the AYP in 2016 was lower than that in 2015. Three combinations, T98S/Z116, T98S/XZ45, and T98S/996, displayed a significant increase in the mean AYP over 2 years and outperformed ZLY819 by producing 15.69, 11.56, and 6.70% more AYP, respectively. Hybrid T98S/XZ45 showed an increase of 10.23% in 2016 but no difference in 2015. The other two hybrid combinations (T98S/ZJZ17 and T98S/Z102) exhibited the same AYP level as ZLY 819. In contrast, the 3-line hybrid rice based on T98A decreased dramatically in AYP due to its abnormal fertility (Table S2; Fig. 4c).

## Discussion

Innovation in the utilization of heterosis techniques is imperative for achieving higher-yield targets in hybrid rice breeding. The 3-line breeding technique opened an era for rice heterosis utilization, and 3-line combination outperformed the same-growth-period inbred conventional rice under the same conditions by 20% in yield (Yuan 1997). Later, the 2-line breeding technique relying on P/TGMS lines was successfully established (Shi 1985), and it has prompted the rapid development of higher-yield super rice breeding because P/TGMS rice has a broad selection of resources (Yuan 1997). Accordingly, several elite super 2-line hybrid rice lines such as Peiai-64S/93-11(Liang-You-Pei 9), Y58S/93-11(Y Liang-You No.1), Y58S/Yuan-hui2 (Y Liang-You no. 2), and Y58S/900 (Y Liang-You 900) achieved yield targets of 10.5 t/ha in 2001, 12.0 t/ha in 2004, 13.5 t/ha in 2011, and 15.0 t/ha in 2014 for the Chinese Super Rice Research and Promotion Plan (Yuan 2014; Wu et al 2016).

A moderate parental genetic difference is required in hybrid breeding, and utilizing heterosis among CMS lines (or maintainer lines) and 2-line restorer lines seems promising. Previous studies showed that early-season Indica cultivars from southern China and the varieties originated from Southeast Asian countries were recognized as two heterotic groups, and these lines together were utilized to produce high-yield 3-line hybrid rice (Yuan 1977). Recently, another work using genetic background detection through genome re-sequencing proved that maintainer line V20B was genetically distant to the 2-line restorer line 93-11 (Hu et al. 2014). In another exploration of heterosis between CMS lines and 2-line restorer lines, we investigated the yield factors from combinations of T98A crossed with 2-line early-season restorer lines such as XZ45, Z116, and Z996 and found that the combinations presented a panicle and grain structure better than that of the commonly used hybrid rice ZLY819 (Table S2; Fig. 4). Disappointingly, however, these combinations could not be fertilized and fruited due to the infertility of the male parents. Our strategy of transforming a maintainer line into a P/TGMS line can make full use of their heterosis. The fact that we identified TGMS line T98S from T98B produced by ^60^CO-γ irradiation treatment (Fig. 1) and succeeded in obtaining high-yield combinations from T98S crossed with 2-line restorer lines opens a gate for diversifying the female lines of rice hybrids (Fig. 4). This outcome is of great importance in solving the problem in which in 3-line rice hybrid systems, the wild-abortive germplasm accounts for more than 60% of the CMS germplasm background (Ding et al. 2007), and only very limited germplasm types can restore fertility when crossed with an abortive CMS line. When more lines can be converted to PTGMS lines in 2-line systems, the major constraint of 3-line hybrid rice systems having a narrow genetic background is addressed, thus conferring great potential in advancing genetic gains in hybrid rice breeding.

A key point in this strategy is to choose a maintainer line with agronomic traits that are as desirable as possible for converting into a PTGMS line. In general, strong general combining ability should be highlighted first, which will maximize the selection probability for elite combinations (Chen et al. 1996). Maintainer line T98B is an ideal germplasm with a strong combining ability derived from core parent V20B (Deng et al. 2001), and it has been employed to develop more than 60 combinations via its CMS line T98A (http://www.ricedata.cn/variety/). In this study, six combinations based on T98S had a high seed-setting rate above 80.00% in 2015, three of which showed a mean yield increase of 6.70–15.69% compared with ZLY819 in 2015 and 2016 (Table S2; Fig. 4), suggesting that T98S has potential for generating new high-yield combinations. Optimal flowering habits are another trait that is critical for enhancing the productivity of hybrid rice seeds (Chen et al. 1996). T98A/T98B has a good flowering habit, and the hybrid seed production based on T98A can reach a high level of 4.50–5.25 t/ha (Deng et al. 2001). In our study, the TGMS line T98S demonstrated a forenoon flowering rate of 75.92% and a high exerted-stigma flower rate of 64.59%, which was considerably better than that in T98A (Fig. 3b, c).

Identifying a PTGMS line in the field is another key step in this strategy that involves continuous observations of fertility under varied temperatures and photoperiods. Our field experiment was carried out in Changsha, China, from early May to late October, with temperatures ranging from 22.0 to 35.0 °C and photoperiods varying from 12.0 to 14.0 h (Fig. 1). Such conditions are highly suitable for screening PTGMS lines. T98S underwent alternations in fertility at least twice. The first alternation from fertility to sterility occurred from May 10th to 20th, and the second shift from sterility to fertility occurred from September 10th to 25th. Both periods involved violent fluctuations in temperature. Ultimately, through a fixed environment in a chamber, we proved that T98S was a forward-type TGMS line (FTGMS) with sterility under high temperatures but fertility at lower temperatures (Fig. 1). In fact, most TGMS lines in practice, such as Y58S and Guang-Zhan 63S, are FTGMS lines (Deng 2005; Yang et al. 2002). FTGMS lines produce hybrid rice seeds safely over an extensive area during a long period since they can maintain sterility from early June to late October in southern China. However, the reverse-type TGMS line (RTGMS) exhibits sterility at lower temperatures but fertility at higher temperatures (Yang and Zhu 1996) and is quite unfavorable for producing F_1_ seeds in early spring at lower latitudes or in summer at higher latitudes. Thus, from this perspective, T98S is a very productive FTGMS line. Furthermore, due to T98S being equal to T98A in genetic background and basic agronomic traits, we considered T98S to be a good replacement for T98A in producing hybrid seeds at a lower cost and with easier seed field management.

Given that foreign P/TGMS plants were probably mixed in with the mutagenesis population, it was necessary to validate whether T98S mutated from T98B. To this end, we compared T98S with T98B both at the morphological level and at the genetic level. Investigation of the morphological specificity revealed that T98S was very similar to T98B in plant shape, and they showed identical agronomic characteristics including panicle length, grain number per panicle, and flowering habits (Table S1; Fig. 3). Similar results were observed in paired combinations based on crossing T98S and T98A with the same female parent (Table S2; Fig. 4). To further confirm that T98S was derived from T98B, we carried out a genetic background test using 48 parental polymorphism SSR markers (Lin et al. 2016), and the results (Fig. 2) also supported that T98S was a single-locus mutant from T98B.

A series of studies showed that P/TGMS mutants are likely to be obtained by point mutation or indel mutation, and thus, a few *p/tgms* genes have been discovered located on various chromosomes. For instance, the first documented PTGMS line was identified as a natural mutant among an open field of the rice variety “Nongken58” in 1973, and it was later confirmed by Mendelian genetic analysis that the photosensitivity of lines is controlled by a recessive male-sterile allele in rice (Shi 1985). Many subsequent publications supported Shi’s conclusion, such as those showing that *Sokcho-MS* (Lee et al. 2005) and *G20S* (Liu et al. 2010) were spontaneously originated from mutations on chromosomes 5 and 10, that *TGMS-VN1* (Dong et al. 2000) and *csa* (Zhang et al. 2010) were obtained by gamma irradiation on chromosome 5 and chromosome 1, and that *0A15-1* was developed via cell culture on chromosome 3 (Wang et al. 2004). Recently, P/TGMS lines were successfully directionally cultivated from *TMS5* by genome editing with a CRISPR/Cas9-mediated system (Zhou et al. 2016), which will allow the elite maintainer lines to be converted into P/TGMS lines effectively. Although CRISPR and P/TGMS allele conversions with the aid of MAS are alternative options in generating new P/TGMS germplasms and driving faster genetic gain in 2-line rice hybrid systems; our approach is less time-consuming and does not involve any genetic transformation process. Thus, it can add value to the exploration of better P/TGMS lines.

Fundamental research on map-based cloning and functional analysis of the *tgms* gene in T98S should also be conducted. Additional applied work on establishing a method of marker-assisted breeding by converting T98S together with screening TGMS plants for a proper fertility alteration temperature should be undertaken in the near future.

## Conclusion

This paper presents a strategy for generating the TGMS line T98S from the rice maintainer line T98B. T98S inherited the basic genetic background and maintained the elite traits of T98B. T98S male sterility is controlled by a recessive nuclear gene. The strategy reported here is quite user friendly when working with 2-line hybrid system restorer lines for developing hybrid rice with better yield potential. Thus, the strategy of converting a maintainer line into a P/TGMS line is practical for the development of hybrid rice with more desirable heterosis.

## Electronic supplementary material

ESM 1 (DOC 161 kb)

ESM 2 (DOC 5.18 mb)

ESM 3 (DOC 31.0 kb)

ESM 4 (DOC 81.0 kb)
