# Supplementary material for: Conversion of a rice CMS maintainer into a photo- or thermo-sensitive genetic male sterile line
Source: Mol Breed. 2018 Apr 18;38(5):56. doi: 10.1007/s11032-018-0805-2 (PMC5906493; doi:10.1007/s11032-018-0805-2)
Supplement: Supplementary file 1 — (DOC 161 kb) [file 11032_2018_805_MOESM1_ESM.doc]

| 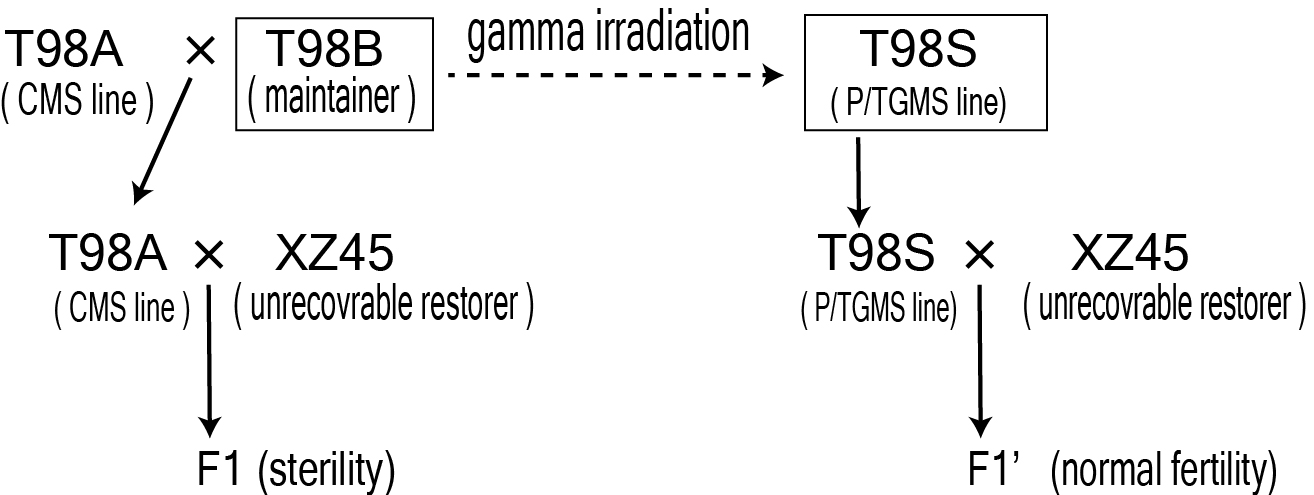 |
| --- |
| **Fig. S1** A model for a strategy utilizing heterosis between maintainer lines  and 2-line restorer lines  The CMS line T98A, maintainer line T98B and P/TGMS line T98S present very similar genetic backgrounds. Xiang-Zao 45 (XZ45) is a 2-line restorer line that introduces defects in recovering CMS line T98A; therefore, the combination of T98A/XZ45 seems useless for developing sterility in the T98B line. However, T98S converted from maintainer line T98B by gamma irradiation can be fully recovered by XZ45, making T98S/XZ45 a potentially useful combination. |
