# Supplementary material for: Conversion of a rice CMS maintainer into a photo- or thermo-sensitive genetic male sterile line
Source: Mol Breed. 2018 Apr 18;38(5):56. doi: 10.1007/s11032-018-0805-2 (PMC5906493; doi:10.1007/s11032-018-0805-2)
Supplement: Supplementary file 2 — (DOC 5.18 mb) [file 11032_2018_805_MOESM2_ESM.doc]

| 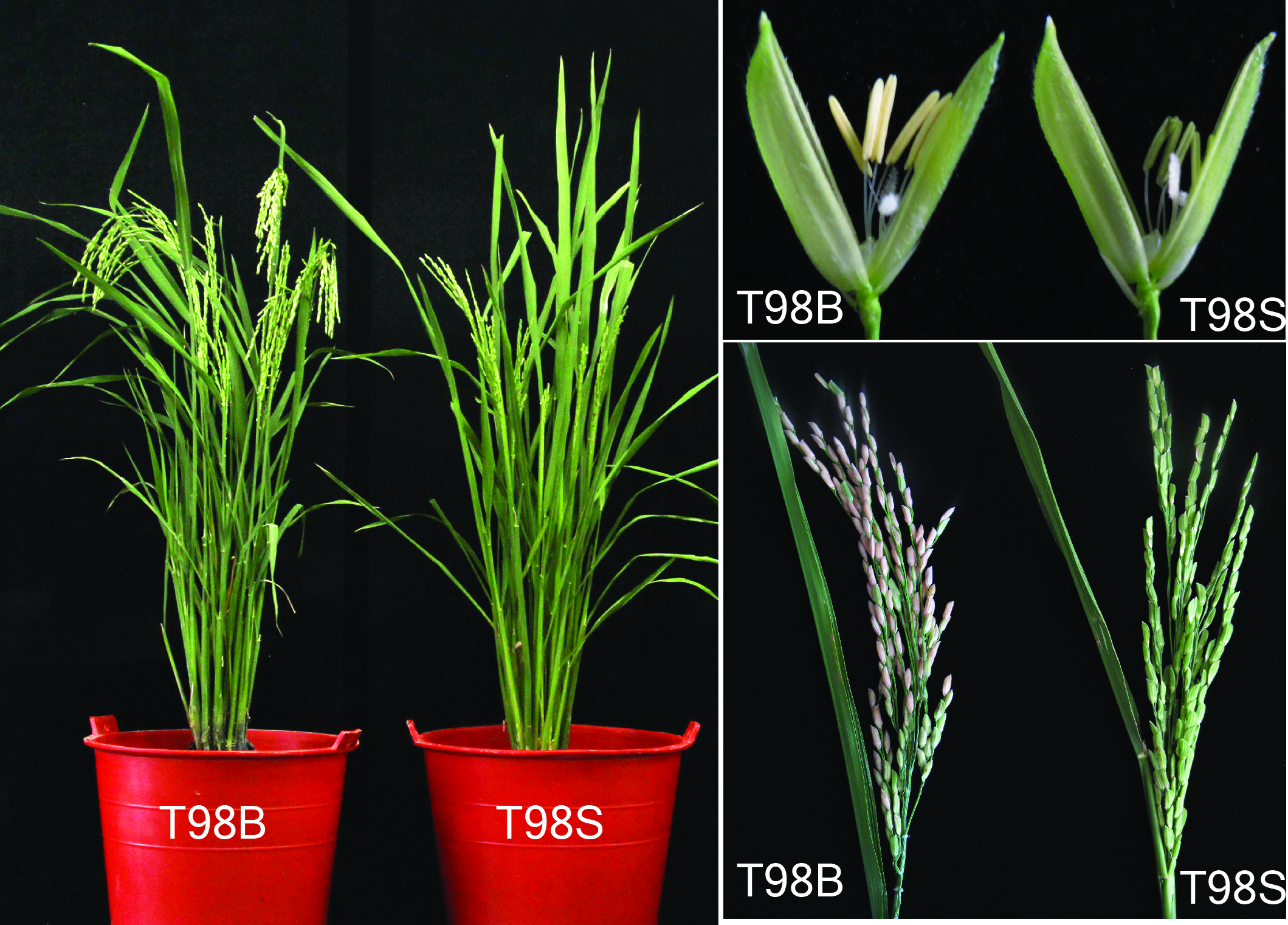 |
| --- |
| **Fig. S2** Morphological observation of T98S compared with T98B  T98S and T98B were observed in 2014 (sown on May 24^th^) in Changsha, China. They appeared alike in plant shape except for fertility traits. T98B normally presented as fertile, while T98S was male sterile. |
|
