# Supplementary material for: Conversion of a rice CMS maintainer into a photo- or thermo-sensitive genetic male sterile line
Source: Mol Breed. 2018 Apr 18;38(5):56. doi: 10.1007/s11032-018-0805-2 (PMC5906493; doi:10.1007/s11032-018-0805-2)
Supplement: Supplementary file 3 — (DOC 31.0 kb) [file 11032_2018_805_MOESM3_ESM.doc]

**Table S1** Inheritance of the TGMS gene in T98S

| Cross | Generation | Total no.  of plants | No. of  fertile plants | No. of  TGMS plants | χ2_(3:1)_ | P |
| --- | --- | --- | --- | --- | --- | --- |
| T98S/T98B | F1 | 43 | 43 | 0 | - | - |
|  | F2 | 437 | 338 | 99 | 1.16 | 0.20～0.30 |
| T98S/XZ45 | F1 | 50 | 50 | 0 | - | - |
|  | F2 | 3016 | 2303 | 713 | 2.90 | 0.05～0.10 |

Data were obtained by a field fertility observation under high-temperature conditions.
