# Supplementary material for: Conversion of a rice CMS maintainer into a photo- or thermo-sensitive genetic male sterile line
Source: Mol Breed. 2018 Apr 18;38(5):56. doi: 10.1007/s11032-018-0805-2 (PMC5906493; doi:10.1007/s11032-018-0805-2)
Supplement: Supplementary file 4 — (DOC 81.0 kb) [file 11032_2018_805_MOESM4_ESM.doc]

**Table S2 Yield factors for combinations of T98S and T98A crossed with 2-line restorer lines**

| Year and location | Combination | WGP  (d) | PH  (cm) | EPP | GNP | SSR  (%) | TGW  (g) | AYP  (g) | AYP increase  (%) |
| --- | --- | --- | --- | --- | --- | --- | --- | --- | --- |
| 2015,  Changsha, China | ZLY819 (CK) | 107.0±1.0 | 102.6±2.5 | 8.4±0.7 | 117.4±6.3 | 78.03±5.28 | 24.3±0.6 | 19.86±0.46 | 0.00% |
|  | T98S/XZ45 | 107.7±0.6 | 100.7±3.1 | 8.6±0.6 | 142.2±6.8* | 84.47±5.20* | 24.4±0.6 | 21.48±0.57* | 8.16% |
|  | T98A/XZ45 | 107.0±1.0 | 103.1±3.9 | 8.9±0.6 | 136.9±6.3* | 26.32±3.42** | 24.5±0.5 | 4.07±0.37** | -79.51% |
|  | T98S/ZJZ17 | 108.3±1.1 | 98.4±3.4 | 7.9±0.4* | 119.4±6.2 | 82.47±4.84 | 25.7±0.5* | 19.85±0.41 | -0.05% |
|  | T98A/ZJZ17 | 108.7±0.6 | 96.2±2.8* | 7.5±0.4* | 121.7±5.5 | 10.82±2.46** | 25.4±0.5* | 1.09±0.23** | -94.51% |
|  | T98S/Z116 | 108.7±0.6 | 105.0±3.9 | 9.4±0.7* | 135.2±4.9* | 82.66±6.32* | 23.6±0.5* | 22.72±0.57* | 14.40% |
|  | T98A/Z116 | 108.0±1.0 | 103.4±3.5 | 9.1±0.5* | 139.7±5.9* | 20.21±5.57** | 24.0±0.5 | 4.14±0.19** | -79.15% |
|  | T98S/Z102 | 106.3±1.1 | 102.8±3.4 | 7.8±0.4* | 144.6±8.4* | 81.25±5.43 | 22.9±0.4* | 20.18±0.49 | 1.61% |
|  | T98A/Z102 | 105.7±0.6 | 99.6±3.9 | 8.1±0.6 | 139.4±7.3* | 24.74±4.82** | 23.1±0.4* | 3.82±0.30** | -80.77% |
|  | T98S/Z143 | 108.7±0.6 | 97.6±2.9 | 8.7±0.7 | 118.7±6.4 | 81.55±4.21 | 24.8±0.5 | 20.04±0.42 | 0.91% |
|  | T98A/Z143 | 108.6±1.5 | 99.6±3.2 | 8.8±0.4 | 112.4±6.3 | 8.20±1.77** | 24.6±0.6 | 1.17±0.36** | -94.11% |
|  | T98S/Z996 | 108.0±1.0 | 107.8±3.8* | 7.6±0.6* | 152.2±7.3* | 81.31±5.87 | 23.2±0.4 | 21.09±0.35* | 6.19% |
|  | T98A/Z996 | 108.7±0.6 | 109.6±4.1* | 7.9±0.5* | 147.5±6.7* | 13.72±2.19** | 23.5±0.6 | 4.18±0.43** | -78.95% |
| 2016,  Changsha, China | ZLY819 (CK) | 105.7±0.6 | 105.4±3.4 | 8.3± 0.4 | 109.3±4.7 | 73.50±3.28 | 24.3±0.6 | 18.85±0.51 | 0.00% |
|  | T98S/XZ45 | 105.0±1.0 | 101.4±3.7 | 8.3±0.7 | 125.4±5.3* | 76.22±3.75 | 24.7±0.5 | 21.67±0.53* | 14.96% |
|  | T98A/XZ45 | 105.7±0.6 | 105.9±4.2 | 8.6±0.6 | 130.7±6.2* | 20.02±2.51** | 24.9±0.4 | 3.27±0.33** | -82.65% |
|  | T98S/ZJZ17 | 105.7±0.6 | 97.8±3.8* | 8.5±0.4 | 101.3±3.8 | 73.53±3.82 | 25.3±0.4* | 18.76±0.67 | -0.48% |
|  | T98A/ZJZ17 | 106.3±1.1 | 96.9±3.1* | 8.7±0.7 | 102.8±4.2 | 7.55±2.47** | 25.6±0.4* | 1.15±0.22** | -93.90% |
|  | T98S/Z116 | 106.0±1.0 | 107.2±4.4 | 8.9±0.4 | 120.6±4.7* | 73.83±3.88 | 23.8±0.6 | 22.05±0.61* | 16.98% |
|  | T98A/Z116 | 106.0±1.0 | 106.4±3.7 | 8.4±0.7 | 125.7±4.4* | 16.14±5.06** | 24.2±0.4 | 3.07±0.37** | -83.71% |
|  | T98S/Z102 | 105.7±0.6 | 104.8±4.7 | 7.8±0.4* | 133.4±4.4* | 77.74±3.67 | 22.2±0.3* | 20.78±0.49* | 10.24% |
|  | T98A/Z102 | 105.7±0.6 | 106.6±4.7 | 7.9±0.5 | 127.9±4.3* | 16.76±2.41** | 22.5±0.5* | 3.1±0.41** | -83.55% |
|  | T98S/Z143 | 106.0±1.0 | 98.2±3.3* | 8.4 ±0.7 | 115.2±3.9 | 76.43±3.85a | 24.1±0.4 | 19.35±0.72 | 2.65% |
|  | T98A/Z143 | 106.7±0.6 | 101.4±3.1* | 8.6 ±0.6 | 118.7±4.5 | 11.14±1.57** | 24.6±0.5 | 1.3±0.44** | -93.10% |
|  | T98S/Z996 | 107.3±1.1 | 113.3±3.7* | 8.0±0.4 | 133.4±5.8* | 75.81±4.22 | 23.4±0.4* | 20.21±0.49* | 7.21% |
|  | T98A/Z996 | 107.0±1.0 | 114.6±4.7* | 7.7±0.7* | 136.2±5.3* | 11.67±3.28** | 23.8±0.5 | 3.76±0.46** | -80.05% |

WGP, whole growth period; PH, plant height; EPP, effective panicle per plant; GNP, grain number per panicle; SSR, seed-setting rate; TGW, thousand-grain weight; AYP, actual yield per plant (AYP). Values are mean±sd. * and ** indicate p<0.05 and p<0.01, respectively.
